# The Effects of Electric Fields on Protein Phase Behavior and Protein Crystallization Kinetics

**DOI:** 10.1021/acs.jpclett.4c01744

**Published:** 2024-08-01

**Authors:** D. Ray, M. Madani, J. K. G. Dhont, F. Platten, K. Kang

**Affiliations:** † Institute of Biological Information Processing IBI-4, Forschungszentrum Jülich, 52428, Jülich, Germany; ‡ Faculty of Mathematics and Natural Sciences, Heinrich Heine University Düsseldorf, 40225 Düsseldorf, Germany; § Solid State Physics Division, Bhabha Atomic Research Centre, Trombay, Mumbai 400085, India

## Abstract

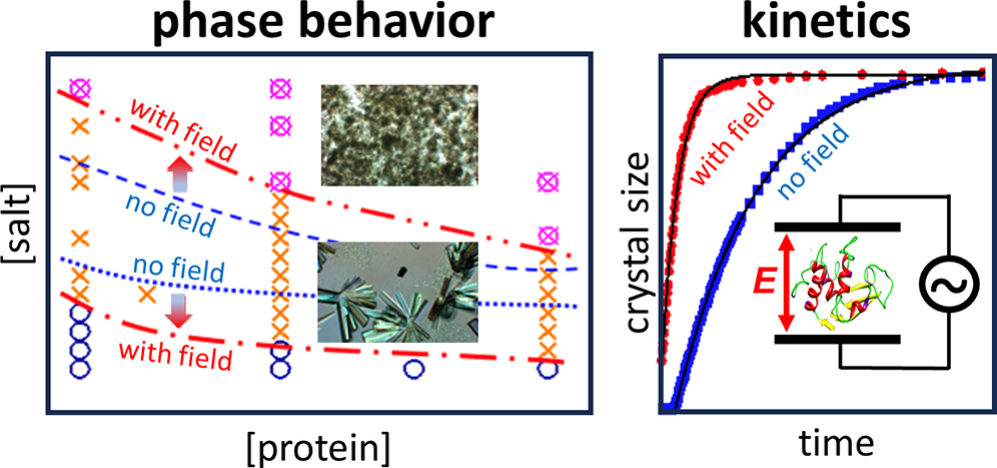

We experimentally studied the effects of an externally applied electric field on protein crystallization and liquid–liquid phase separation (LLPS) and its crystallization kinetics. For a surprisingly weak alternating current (AC) electric field, crystallization was found to occur in a wider region of the phase diagram, while nucleation induction times were reduced, and crystal growth rates were enhanced. LLPS on the contrary was suppressed, which diminishes the tendency for a two-step crystallization scenario. The effect of the electric field is ascribed to a change in the protein–protein interaction potential.

Proteins are fundamental to the structure and function of living organisms. They can undergo transitions between different states of organization, ranging from soluble monomers, over disordered states (including gels, clusters, amorphous aggregates, as well as liquid–liquid phase separation (LLPS)) to ordered structures (like crystals and fibrils).^1−6^ The formation of these states depends on the delicate balance of excluded volume interactions, electrostatic repulsion, and short-range attraction,^7−12^ which can be tuned by control variables such as protein concentration, temperature, pH, ionic strength, and additives.^13−15^ Protein hydration might also affect phase behavior and interactions.^15−17^ In addition, the specific characteristics of the proteins themselves, such as their size, shape, conformation, and surface properties, also play a crucial role in determining their collective behavior. Controlling the formation of condensed states is crucial for the regulation of cellular processes and the maintenance of cellular homeostasis.^18^ In particular, disruptions in protein phase behavior have been linked to various diseases including neurodegenerative disorders and cancer.^19^ Moreover, protein phase behavior can have significant implications for various fields such as pharmaceuticals, food engineering, and materials science, contributing to the design of novel protein-based materials.^20−22^

Electric fields represent one way to externally affect the protein phase behavior and crystallization kinetics. Electric fields have been employed to heuristically optimize the nucleation and growth processes to obtain diffraction-quality crystals, typically using high field amplitudes (kV/mm) or frequencies (MHz). Both direct current (DC)^23^ and alternating current (AC),^24,25^ as well as pulsed electric fields,^26^ can affect the size, number, and quality of crystals.^27,28^ These effects have, for example, been attributed to locally increased protein and ion concentrations near one of the electrodes in DC fields,^29^ in particular with the large and inhomogeneous fields created by sharp tips,^30^ modifications of the chemical potentials of the liquid and solid states in AC fields,^31^ as well as preferred protein and crystal orientations.^32^ However, in most of these studies, the electric field conditions are not very well-defined.

The changes in the phase behavior due to external electric fields find their origin in the changes in protein–protein interactions. Electric fields may affect interactions between proteins in several ways. For the high electric field strengths used in many of the references mentioned above, dielectric polarization plays an important role. For sufficiently large proteins and strong electric fields, field-induced alignment of single proteins and self-assembled structures may also affect the self-assembly kinetics.^33,34^ However, it is conceivable that for smaller field strengths, typically applied to colloids,^35,36^ other mechanisms might be dominant, which has not been systematically investigated for proteins. The electric field leads to protein-internal stresses through the electric forces onto the charged groups covalently bound to the backbone of the protein. These field-induced stresses can change the conformation of the protein, leading to the exposure of hydrophobic groups to the outer surface of the protein, which enhances the short-range attractive interactions between the proteins. The field-induced deformation of the electric double layer alters the electrostatic protein–protein interactions. The electrostatic forces that affect ions within the double layer are transmitted to the solvent, resulting in electroosmotic flow that induces hydrodynamic interactions. The electrostatic force onto the protein as a whole leads to electrophoretic motion, giving rise to additional hydrodynamic interactions.

The full protein phase diagram and the associated phase transition kinetics in the presence of well-defined electric fields have as yet not been systematically explored. We probe the effect of a relatively weak electric field (V/mm and kHz) on the location of the phase boundaries for crystallization and LLPS, in the salt-versus-protein concentration plane. The kinetics of crystallization, with and without an electric field, is investigated for a given protein and various salt concentrations.

As a model system, we use lysozyme dissolved in acetate buffer (pH 4.5) in the presence of sodium thiocyanate (NaSCN). Specific interactions of thiocyanate (SCN) with lysozyme lead to additional protein–protein attractions, which lowers the lysozyme solubility.^37^ The choice of this salt is motivated by the relatively low salt concentrations where crystallization and LLPS are observed as compared to, for example, sodium chloride.^38^ These low salt concentrations are necessary to avoid electric-field-induced heating of the protein solution. AC electric fields are applied using a function generator (Siglent SDG830), in combination with a home-built optically transparent indium–tin oxide (ITO)-coated glass electric cell, with a distance between the electrodes of 160 μm. We use an electric field condition (frequency 1 kHz and field strength 6 V/mm) similar to those to which amorphous protein aggregates have been shown to respond.^39^ The morphologies that occur in protein solutions at ambient temperature are obtained by polarized optical microscopy (Zeiss Axiovert 40CFL with an AxioCam Color CCD camera). Detailed experimental and analysis procedures are given in the Supporting Information (SI), which includes refs (40 and 41.)

Figure 1a shows typical micrographs of the three major states, a homogeneous solution, crystals in monoclinic form, and LLPS, where LLPS is metastable with respect to crystallization.^42,43^ The different states are identified by their characteristic microscopic morphologies: the absence of micron-sized objects; the presence of crystals (if formed during the experimental observation period of up to 72 h); or the occurrence of a sharp increase of the turbidity^13^ followed by droplet or domain formation and coarsening, respectively. The phase diagram in the protein versus salt concentration plane without the electric field is given in Figure 1b with phase boundaries shown as dashed blue lines. Figure 1c shows the phase diagram in the presence of the electric field, where phase boundaries are indicated by the dashed-dotted red lines. For comparison, the phase boundaries in the absence of the electric field are also shown. The two arrows indicate the field-induced shift of the phase boundaries, which is seen to be quite significant. The electric field promotes crystallization at the expense of both the homogeneous liquid and LLPS.

**Figure 1:**
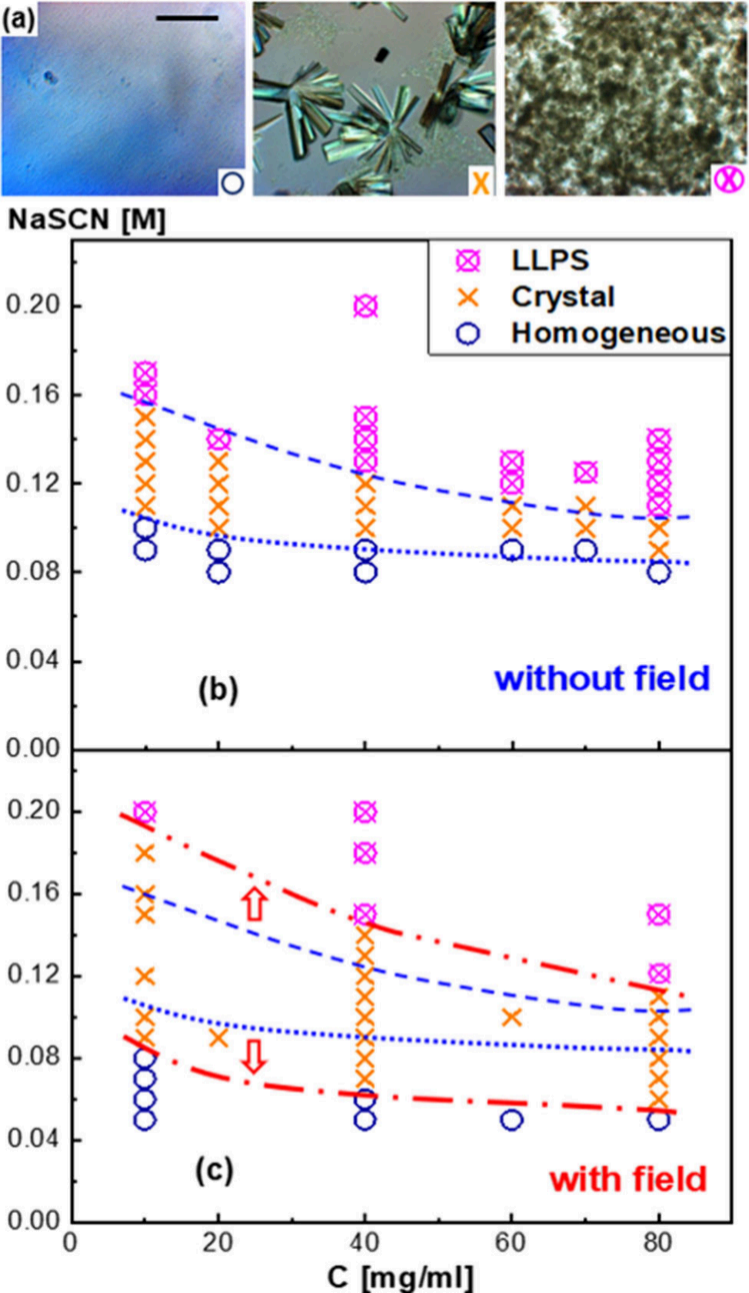
(a) Typical depolarized light-microscopy images. From left to right: homogeneous solution, protein crystals, and metastable LLPS. The scale bar is 200 μm. The phases are indicated by the symbols shown in the right-bottom corner of the images: blue circles, orange crosses, and pink crossed circles, respectively. The images refer to samples with a protein concentration *c* = 40 mg/mL and NaSCN concentrations of 0.05, 0.13, and 0.18 M, respectively. (b) Phase diagram (pH 4.5; 50 mM acetate buffer; at (24 ± 1) °C) in the protein versus salt concentration plane, without the electric field. The phase boundaries are indicated by dashed blue lines: the crystallization boundary between the homogeneous solution and the region in which crystal and the liquid phase coexist (lower line) and phase boundary between the crystal-solution coexistence and the metastable LLPS phase (upper line). (c) Phase diagram in the presence of the electric field (frequency 1 kHz and field strength 6 V/mm). The phase boundaries in the presence of the electric field are shown as red dash-dotted lines. The arrows indicate the shifts of phase boundaries due to the electric field.

Upon applying the electric field, the liquid-crystal boundary is shifted toward lower salt concentrations. For a certain solution composition in the crystal-solution coexistence region, this implies an increased (horizontal) distance to the boundary and thus a field-induced increase of the force that drives protein crystallization. In thermodynamic terms, this corresponds to a field-induced increase in the difference between the chemical potential of proteins in solution and in the crystalline phase. On a molecular level, such an increase of the chemical potential correlates with increased attractions between the proteins (see, for example, ref (11)). However, the LLPS boundary is shifted to higher salt concentrations, indicating that attractions are diminished^44^ by the electric field rather than being increased (this will be further discussed below in connection to Figure 4).

An explanation for this apparently contradictory finding is that liquid–liquid phase separation is largely sensitive to overall attractions, averaged with respect to protein orientations, whereas for crystallization, the orientation dependence of the pair-interaction potential is dominant. Proteins are prone to attach to a crystal surface in orientations where the attractions are most pronounced. The field-induced reduction of overall attractions between the proteins diminishes the tendency for a two-step crystallization scenario, where enhanced crystallization results from initially occurring liquid–liquid phase separation.^42,45−47^

That electric field effects can be attributed to changes in the protein–protein pair-interaction potential is further corroborated by crystallization kinetics experiments, as will be discussed below.

The crystallization kinetics is quantified from time-resolved measurements of the length of individual crystals for various NaSCN concentrations at a given protein concentration of 40 mg/mL. As an example, Figure 2 shows selected micrographs and crystal growth curves for a fixed salt concentration in the absence and presence of an electric field. Figure 2a shows images at different times without and with the electric field. These images illustrate the pronounced effect of the electric field on the crystallization kinetics and the size of the crystals in the final state. The length *L* of individual crystals, normalized to their final length *L*_∞_, is plotted as a function of time in Figure 2b. For independently prepared samples, these curves for individual crystals were found to superimpose. Each curve is the average of three independent measurements. The solid lines are fits according to *L*/*L*_∞_ = 1 – exp[−Γ(*t* – *t*_ind_)], where the fitting parameters Γ and *t*_ind_ are the overall crystal growth rate and the induction time, respectively. Similar growth curves have been found in, for example, refs (48 and 49). The inset in Figure 2b shows a blowup of the initial growth curve, together with the same solid line as obtained from the overall fit. The initial growth is accurately captured by the fit. The absolute initial crystal growth rates vary between 1 to 10 μm/min. A pronounced effect of the electric field on the crystallization kinetics is evident, both with respect to nucleation and growth. Similar experiments have been performed at various salt concentrations; details are given in SI.

**Figure 2:**
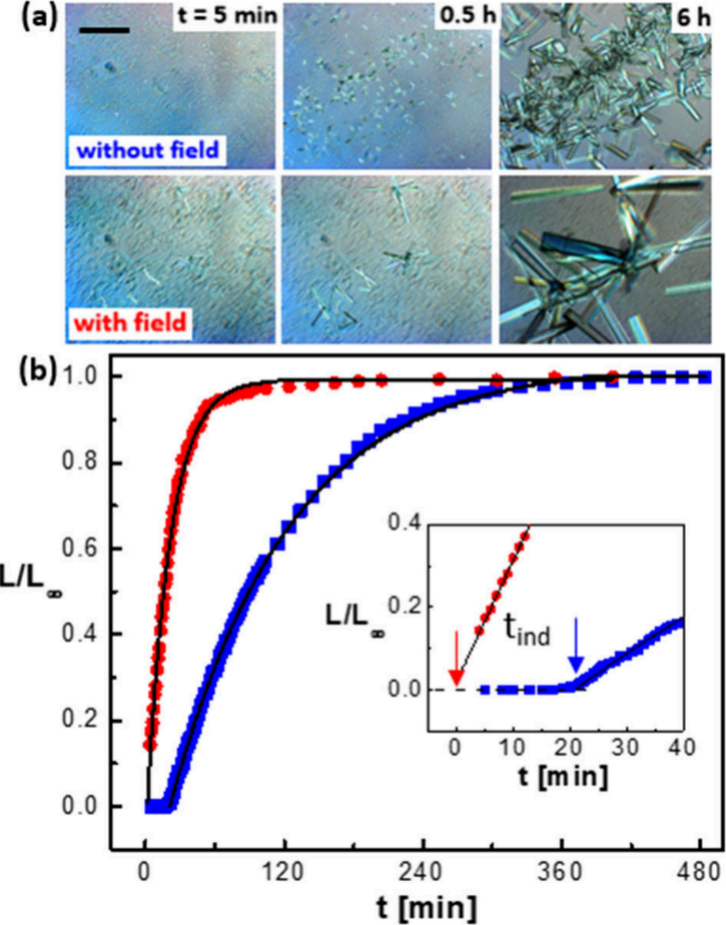
Protein crystallization kinetics in the absence and presence of the electric field, illustrated for a protein solution with protein and salt concentrations of *c* = 40 mg/mL and 0.11 M NaSCN, respectively. (a) Polarized-light micrographs (scale bar: 200 μm) shown at selected different times *t* subsequent to sample preparation in the absence (top) and presence (bottom) of the electric field. (b) Length *L* of a protein crystal, normalized to its final length *L*_∞_, as a function of time *t*, as obtained from a time-lapse series of images, in the absence and presence of the electric field (blue squares and red circles, respectively) and corresponding single-exponential fits (lines). The inset shows a magnified view for small times, where induction times *t*_ind_ are indicated by vertical arrows.

The kinetic parameters of all of the experiments are summarized in Figure 3. The induction time *t*_ind_ and the overall growth rate Γ are plotted as a function of salt concentration in Figure 3, panels a and b, respectively. The induction time is significantly decreased by the electric field, except for higher salt concentrations close to the LLPS boundary. Evidently, the induction time decreases with an increasing salt concentration. Close to the liquid-crystal boundary in the presence of the electric field, the induction time reaches very large values. For salt concentrations within the liquid-crystal coexistence region in the absence of the field, the crystal growth rates are significantly enhanced by the field. The lowering of induction times and the increased crystal growth rates comply with the above inferred increase in the anisotropic attractive interactions between the proteins. Increased attractions lead to enhanced chemical potential, which in turn explains the changes of the induction times and growth rates. The electric field might also lead to a decreased energy barrier against nucleation and enhanced protein attachment rates to the crystal (considerations concerning the effect of the electric field on the chemical potential can be found in the SI, which includes refs (50−53)). Furthermore, for many conditions probed, the electric field yields larger crystals as compared to those obtained in the absence of the field, likely due to larger induction times and smaller growth rates (see SI for more information on crystal sizes).

**Figure 3:**
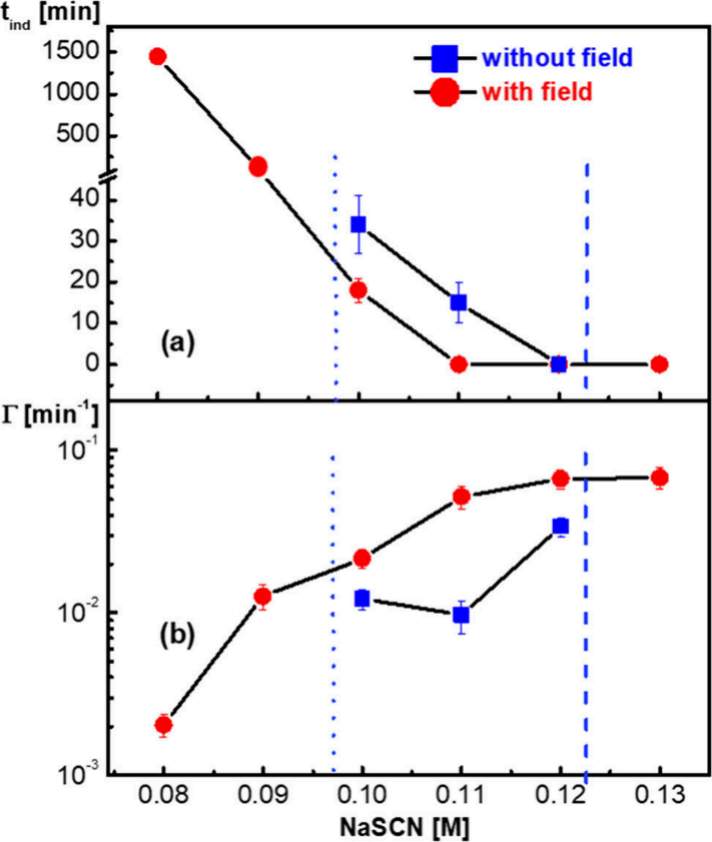
(a) Crystallization induction time *t*_ind_, and (b) the overall crystal growth rate Γ as a function of the salt concentration for a protein concentration of 40 mg/mL. Data are shown in the absence (blue squares) and presence (red circles) of the electric field. Dashed vertical lines indicate the phase boundaries in the absence of the electric field.

The fitting function corresponding to the solid lines in Figure 2b implies that d*L*(*t*)/d*t =* Γ [*L*_∞_ – *L*(*t*)], with a time-independent rate constant Γ, for both the growth curves without and with the electric field. The same functional form of the growth curves indicates that the mechanism for crystallization, and hence the driving force for crystallization are not affected by the electric field. Therefore, the effect of the electric field can be understood entirely in terms of direct interactions between the proteins, while flow and electrophoresis do not play a major role. The driving force for crystallization in the presence of the electric field is therefore proportional to the difference  between the chemical potential of a protein in solution and within the crystal, just as for crystallization in the absence of the electric field. The increasing overall crystal growth rate Γ with the application of an electric field is in accordance with the aforementioned increasing attractions between the proteins.

To further rationalize the field effect on direct interactions, an approximate, semiquantitative measure can be obtained as follows. As discussed above, the effect of the electric field can be attributed to a change in the pair-interaction potential between the proteins. Therefore, we can describe the effect of the field in thermodynamic terms. For near-spherical proteins with orientationally averaged short-ranged interactions, the protein–protein pair-interaction potential can be approximated by a so-called sticky hard sphere potential.^54^ The sticky sphere model corresponds to a square-well system whose well is infinitely narrow and deep in such a way that the second virial coefficient is finite.^54^ This is likely the simplest model for a system with short-range attractions and has been successfully applied to proteins.^55^ Moreover, the corresponding-states law^56^ implies that the model chosen should not matter on the second virial level. To within a second-virial approximation,^57^ the osmotic pressure for such a potential is equal to , where  is the number density of proteins and  is the volume fraction of proteins, with  the volume of a protein. Furthermore, the so-called stickiness parameter  is a measure for the degree of short-ranged attractive interactions between the proteins, in addition to the hard-core excluded volume interactions. A smaller value of  corresponds to a stronger attractive interaction potential. For concentrations corresponding to the spinodal,^58−60^, so that , where  is the volume fraction at the spinodal. In the case the LLPS phase boundary coincides with the spinodal, each protein concentration corresponds to a salt concentration according to the LLPS boundary in Figure 1b,c. The protein concentrations are converted to volume fractions  using the molecular weight 14500 g/mol, and the volume  of lysozyme (see e.g. ref (59)). The stickiness parameter thus obtained is plotted in Figure 4 as a function of the salt concentration. The data points in Figure 4 correspond to numerical values for the protein concentration at the LLPS phase boundary in Figure 1b,c. Note that the values of the stickiness parameter are smaller than its approximate critical value of 0.10,^61,62^ which indicates a close proximity of the LLPS boundary and the liquid–liquid spinodal. The stickiness parameter is larger in the presence of the electric field, indicating an apparent diminishing of the overall orientationally averaged attractive protein–protein forces.

**Figure 4:**
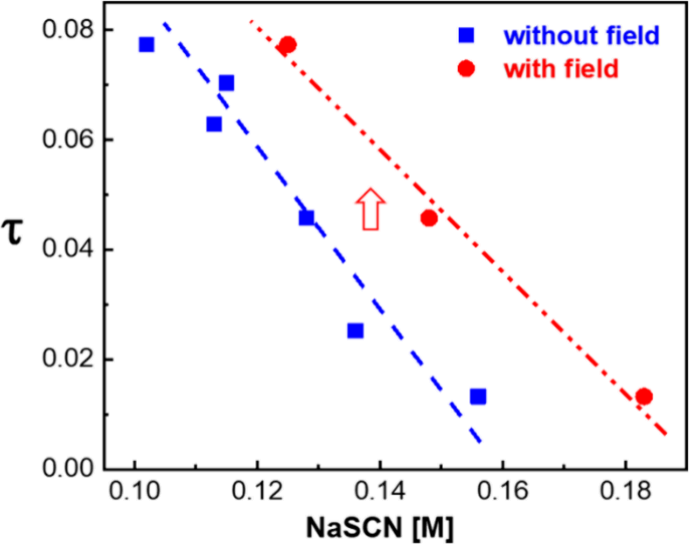
Stickiness parameter τ as a function of salt concentration without the electric field (blue squares) and in the presence of the electric field (red circles), as inferred from the LLPS boundaries in Figure 1b,c. Lines are a guide to the eye. The red arrow indicates the shift in τ in the presence of field.

In summary, we determined the effect of an AC electric field on the protein phase boundaries as a function of protein and salt concentration as well as the crystallization kinetics for a given protein concentration and various salt concentrations. The field conditions (6 V/mm and 1 kHz) are quite mild in comparison to those in earlier studies (typically kV/mm and MHz), where the protein and salt concentrations were not systematically varied. The time dependence of crystal growth rates with and without the electric field is both described by a single-exponential. This indicates that the effect of the electric field is to change the protein–protein interaction potential and that, for example, interactions due to electro-osmotic flow and electrophoresis are insignificant. The liquid-crystal boundary is considerably shifted to lower salt concentrations by the electric field due to field-induced anisotropic attractive interactions. The LLPS boundary, on the other hand, is shifted to higher salt concentrations due to a decrease of overall, orientationally averaged attractions between the proteins, as indicated by the stickiness parameter. These field-induced changes of the protein–protein interaction potential are due to the electrostatic stress that deforms the tertiary structure of the protein and the field-induced deformation of the electric double layer. Since the electric field decreases overall attractions, the two-step scenario for crystallization is suppressed. Computer simulations and NMR experiments in the presence of an electric field would be future pathways to further confirm the above proposed mechanisms for the phase behavior and crystallization kinetics concerning the field-induced protein deformation and the role played by the double layer. In addition, future experiments will be carried out at various field conditions in order to examine whether the observed effects can be enhanced by the field amplitude and in order to decipher the role of different time scales by probing various field parameters.

## Supporting Information Available

Details on Materials & Methods and supporting data (PDF)
Transparent Peer Review report available (PDF)
