# Supplementary material for: The Effects of Electric Fields on Protein Phase Behavior and Protein Crystallization Kinetics
Source: J Phys Chem Lett. 2024 Aug 1;15(31):8108–13. doi: 10.1021/acs.jpclett.4c01744 (PMC11318033; doi:10.1021/acs.jpclett.4c01744)
Supplement: Supplementary file 1 — jz4c01744_si_001.pdf [file jz4c01744_si_001.pdf]

The Effects of Electric Fields on Protein Phase Behavior and Protein Crystallization Kinetics

D. Ray[1,3], M. Madani[2], J.K.G. Dhont[1,2], F. Platten[1,2,*] and K. Kang[1,*]
[1]*Institute of Biological Information Processing IBI-4, Forschungszentrum Jülich, 52428, Germany*
[2]*Faculty of Mathematics and Natural Sciences, Heinrich Heine University Düsseldorf, 40225 Düsseldorf, Germany*
[3]*Solid State Physics Division, Bhabha Atomic Research Centre, Trombay, Mumbai 400085, India*
Email addresses: florian.platten@hhu.de, k.kang@fz-juelich.de

Supporting Information

1. Materials and Methods:

1.1. Sample preparation

Protein and salt are purchased from Sigma-Aldrich as lysozyme from chicken egg white powder (CAS Number: 12650-88-3, Product No. 62971) and reagent grade sodium thiocyanate (NaSCN) (CAS Number: 540-72-7, Product No. S7757), respectively.

Sample solutions are prepared as follows [1]: Lysozyme as a powder is dissolved in buffer (50 mM of sodium acetate buffer adjusted to *p*H 4.5). Then this solution is filtered using a low protein binding filter attachment (Pall Corporation, Acrodisc® Syringe Filters with 0.1 µm Supor® Membrane) at least three times to remove undissolved proteins or aggregates. Similarly, salt was dissolved in the buffer to prepare salt stock solutions. The final concentrations of NaSCN and lysozyme stock solutions are independently measured using a combined density/refractive index measuring system (Anton Paar DMA 4500/RXA 156). Sample solutions with a typical volume of 100 µl are prepared by mixing appropriate amounts of protein and salt stock solutions with buffer. The sample solution is directly transferred onto the microscopy sample cell and monitored. Sample preparation and experiments are performed at room temperature (24 ± 1) °C. Typically, three independently prepared samples are examined for each solution composition and electric field condition.

1.2. Electric-field sample cell

The microscopy sample cell designed for the in-situ observation of electric field effects consists of two thin custom-designed conducting indium-tin-oxide (ITO) glasses (dimension of 40 x70 mm^2) [2]. Before use, the glasses are thoroughly rinsed with a water-ethanol mixture to remove any organic residues. The sample solution is pipetted to the bottom ITO glass that is surrounded with a thin layer of transparent Teflon film spacer (approx. 160 µm). The bottom ITO glass is gently covered with the top ITO glass, and the sample cell is sealed with Teflon tapes. The sample solution squeezed between the two ITO glasses, forms a spherical droplet (with a diameter of about 30 mm) to which the field is applied. To connect the function generator (SDG830 function/arbitrary waveform generator, Siglent), very thin copper tapes (approx.7 mm wide) are attached to the lower outside and upper inside of the ITO glass. The effects of the electric field on the protein solutions are investigated at a field frequency and field strength of 1 kHz and 6 V/mm, respectively. At high frequencies in the MHz regime and high field strength, dielectric polarization of the protein as a whole could lead to dipolar interactions. For the relatively low field strength of 6 V/mm, dielectric polarization of the protein can be neglected. Rather, the polarization of the charge distribution within the electric double layer around a protein molecule will lead to polarization. Due to migration of ions towards the ITO glass electrode surface, the field strength within the bulk of the protein solutions is diminished relative to

the externally applied field strength, a phenomenon that is commonly referred to as electrode polarization. For the present salt concentrations, the distance between the electrodes, and the frequency of 1 kHz, the electric field strength within the bulk protein solution is about 1/3 of the externally applied field strength [3].

1.3. Inverted polarized light microscopy

The optical morphologies are observed using an inverted polarized-light microscope (Axiovert 40CFL) equipped with a CCD camera (AxioCam Color, Carl Zeiss). A low-magnification 10x objective is used to capture a large field of view (1300 x 1029 px^2, corresponding to 821.6 x 650.3 μm^2 in real space). To follow the protein crystallization kinetics, samples with a protein concentration of 40 mg/ml are studied and the micrographs of the morphologies are recorded as time-lapsed images in time intervals of 60 seconds for 6-72 hours, until no further crystal growth is observed.

1.4. Determination of the state diagram

After sample preparation, the morphologies are monitored for a few hours. For the conditions probed, crystals typically form within minutes to hours. The condensed state of the protein solution is assigned based on the microscopic morphology: absence of micron-sized objects (homogeneous solution); crystals in monoclinic form (crystal-solution coexistence), as expected to occur in the presence of NaSCN [4]; and cloudiness followed by droplet formation or domain formation and coarsening (metastable LLPS), as expected for phase separation [5,6].

1.5. Determination of the kinetic parameters characterizing protein crystallization

To characterize the protein crystallization kinetics, a time-lapse series of images is analyzed in the following manner: once the first crystal is observed in the field of view, its growth is followed. In most cases, the orientation of the crystal can be clearly identified in the micrographs, as they typically grow in the observation plane to dimensions much larger than the depth of the cell. The characteristic length of the crystal, L, is determined as a function of time until the crystal has reached its final length, L_∞. Initially, the length increases linearly in time, according to

$$L(t) = G\,(t - t_{\mathrm{ind}}),$$

where t denotes the time after the start of the experiment, $t_{\mathrm{ind}} \geq 0$ is the crystallization induction time [7], and G is the (initial) growth rate of the (010) polar face of lysozyme [8]. The parameters t_{ind} and G are retrieved from linear fits to the $L(t)$ data**.** Results obtained from independent measurements are averaged. Although a certain minimum size (i.e. a certain minimum growth of the crystal) is required such that it can be observed microscopically, the crystallization induction time is expected to be inversely proportional to the nucleation rate [7]. The growth of the $(0\bar{1}0)$ face proceeds much slower and has not been determined here. To describe $L(t)$ for all times, an empirical equation can be used:

$$L/L_\infty = 1\text{-}\exp[-\Gamma(t\text{-}t_{\mathrm{ind}})],$$

where Γ is the overall crystal growth rate. The time-dependent decrease of the (overall) growth rate can be ascribed to the reduced supersaturation during the later stages of the growth and possibly to the presence of additional crystals that have formed later.

2. Supporting data:

2.1. Protein crystallization without and with electric field

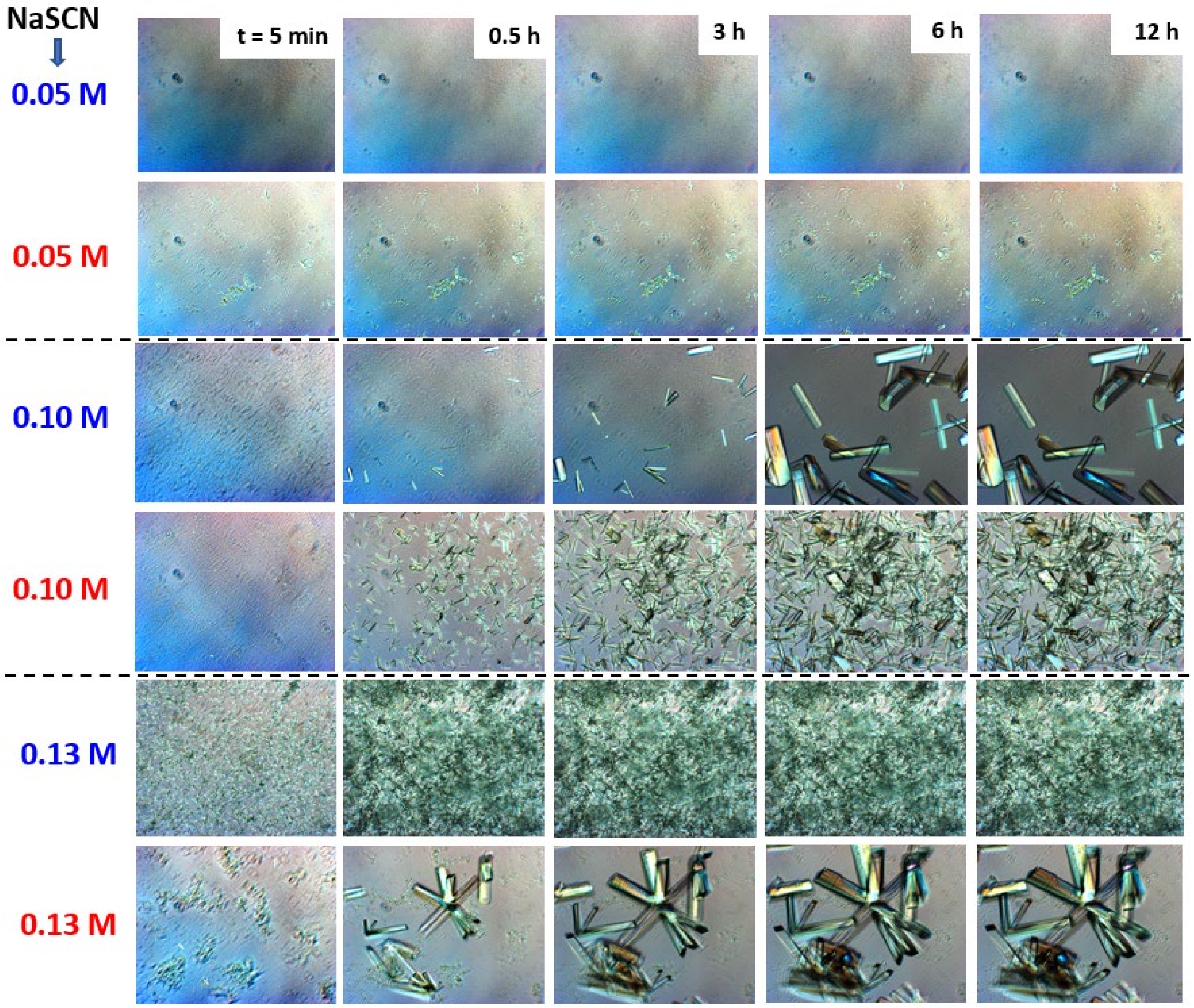

Fig. S1. Time-resolved snapshots from inverted optical microscopy of lysozyme (40 mg/ml) for various NaSCN concentrations in the absence (blue color) and presence (red color) of the electric field. The label in each image depicts the time subsequent to sample preparation.

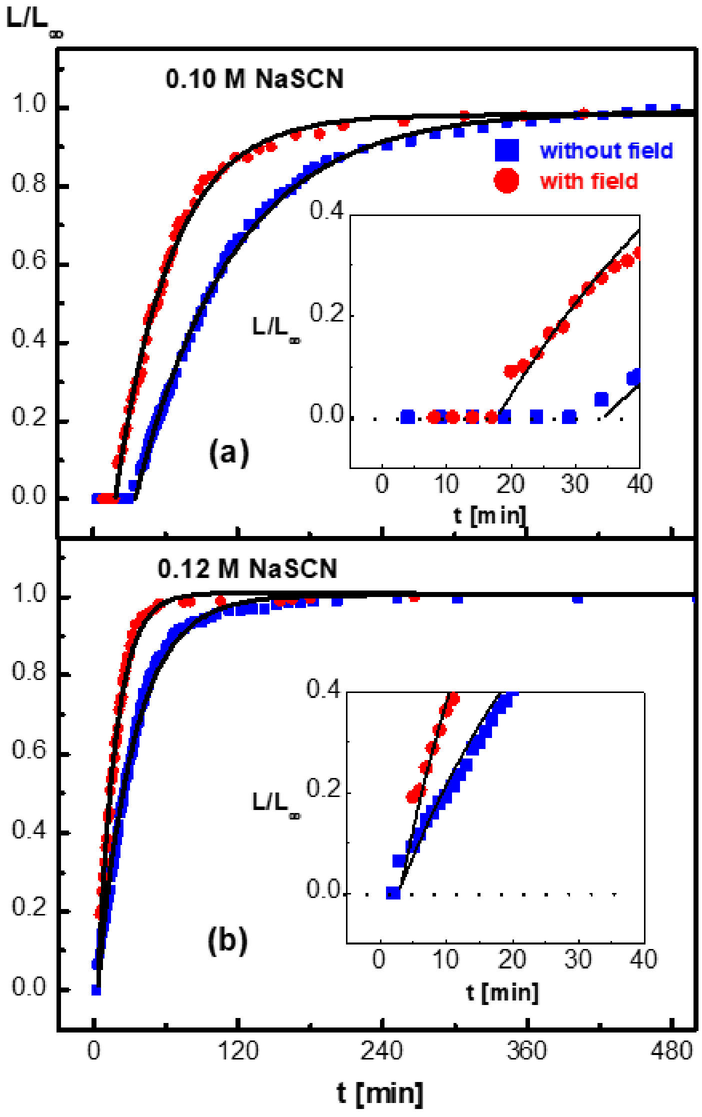

Fig. S2. Normalized growth curves, L(t)/L∞, of lysozyme crystals (40 mg/ml) for various NaSCN concentrations, (a) 0.10 M and (b) 0.12 M in the absence and presence of the electric field. Insets show magnified views for short times.

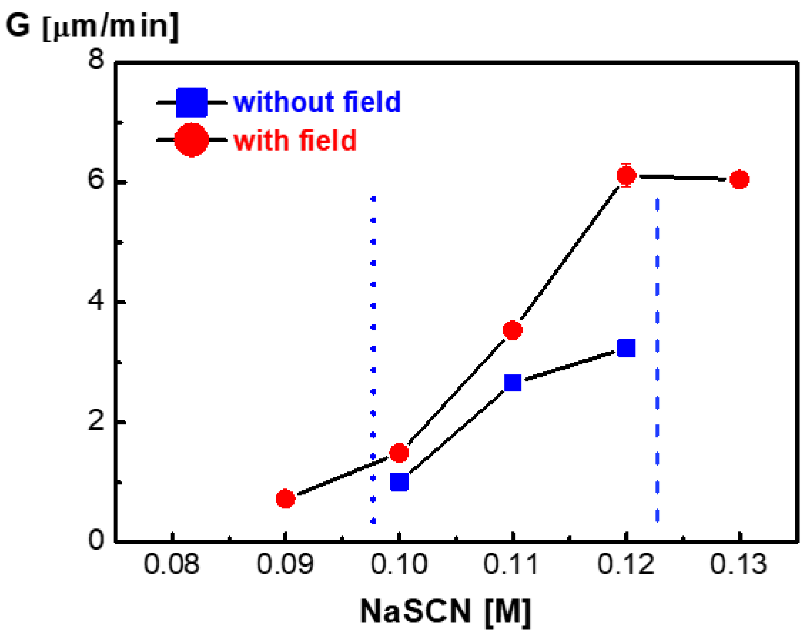

FIG. S3. The initial crystal growth rate G as a function of the salt concentration for a lysozyme concentration of 40 mg/ml in the absence and presence of the electric field. Dashed vertical lines indicate state boundaries in the absence of the electric field.

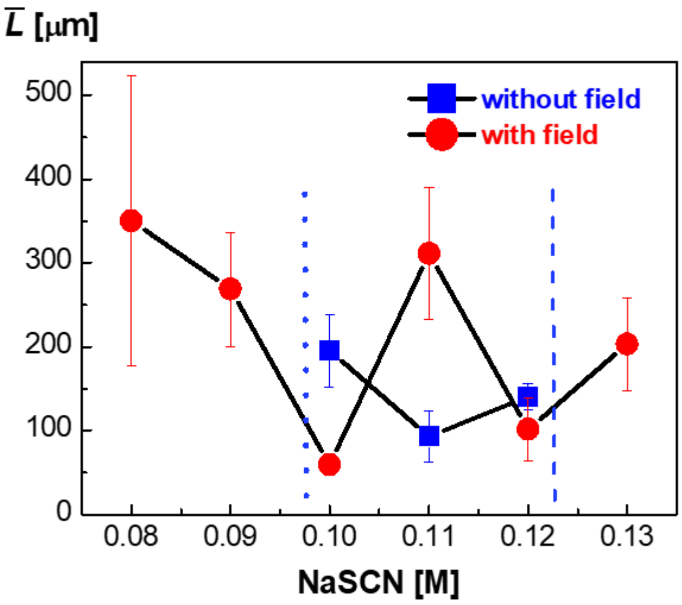

FIG. S4. Mean final length of lysozyme crystals, $\overline{L}$, as a function of the salt concentration for a lysozyme concentration of 40 mg/ml in the absence and presence of the electric field. Error bars indicate the standard deviation. Dashed vertical lines indicate state boundaries in the absence of the electric field.

2.2. Chemical potential in the absence and presence of the electric field:

In the absence of an electric field, the difference in chemical potential of the protein in solution and in the crystal, $\Delta\mu$, can be written as follows:

$$\beta\Delta\mu = \beta\Delta\mu_0 = \ln\left(\frac{c}{c_{\text{eq}}}\right)$$

with the protein concentration c, the solubility c_{eq}, and the inverse thermal energy β. The subscript 0 denotes the absence of an electric field. Note that this equation is only valid in sufficiently dilute solutions, as assumed for the present crystallization experiments. In this case, the effect of the protein-protein interactions on $\Delta\mu$ is only reflected in the value of c_{eq}. Stronger attractions lead to smaller solubility. The solubility of lysozyme in NaSCN has been determined in ref. [4]. These literature data could be empirically interpolated using a power-law function in order to obtain c_{eq} at all salt concentrations studied here. Then the equation given above is used to compute $\beta\Delta\mu$, as shown in Fig. S5 (blue squares).

It is conjectured that the application of an external electric field to the solution leads to field-induced interactions which in turn effectively contribute to the chemical potential. As a consequence, if the electric field is applied, one could assume

$$\Delta\mu = \Delta\mu_0 + \Delta\mu_{\text{E}}$$

with a field-dependent term $\Delta\mu_{\text{E}}$. In the present case, i.e., only one field condition, it can be assumed to be constant. The effective field contribution to the chemical potential can be estimated in the following way. The experimental state diagram in the presence of an electric field (Fig. 1c in the main text) can be compared with $\Delta\mu_0$. Looking at a fixed protein concentration, say 40 mg/ml, protein crystals are formed spontaneously at a lower salt concentration in the presence of the field than in its absence. However, assuming that the actual $\Delta\mu$ necessary for spontaneous crystallization is the same regardless of the presence of the electric field, one can estimate: $\beta\Delta\mu_{\text{E}} \approx 0.7$. The resulting values of $\Delta\mu$ are shown as red circles in Fig. S5.

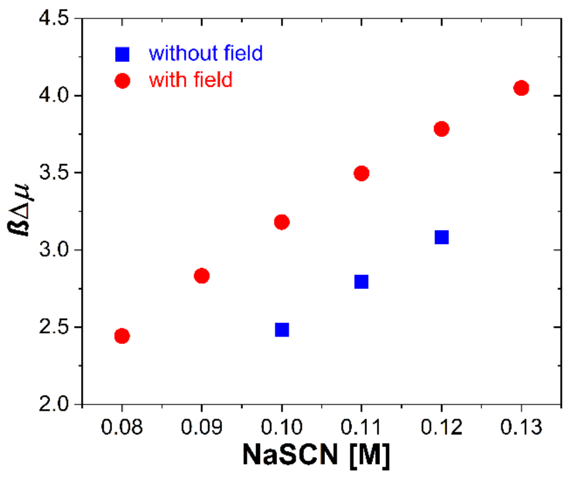

Fig. S5. Chemical potential difference $\Delta\mu$ as a function of the salt concentration for a protein concentration of 40 mg/ml.

2.3. Protein crystallization kinetics:

Since $\Delta\mu$ is available for all conditions for which the crystallization kinetics has been studied (cf. Fig. S5), the kinetic parameters, induction time t_{ind} (cf. Fig. 3(a) in the main text) and initial crystal growth rate G (Fig. S3) can now be represented as a function of $\Delta\mu$ (instead of salt concentration), such that their dependence on $\Delta\mu$ can be analyzed theoretically. As will be shown below, these dependencies can be rationalized. The results are summarized in Fig. S6.

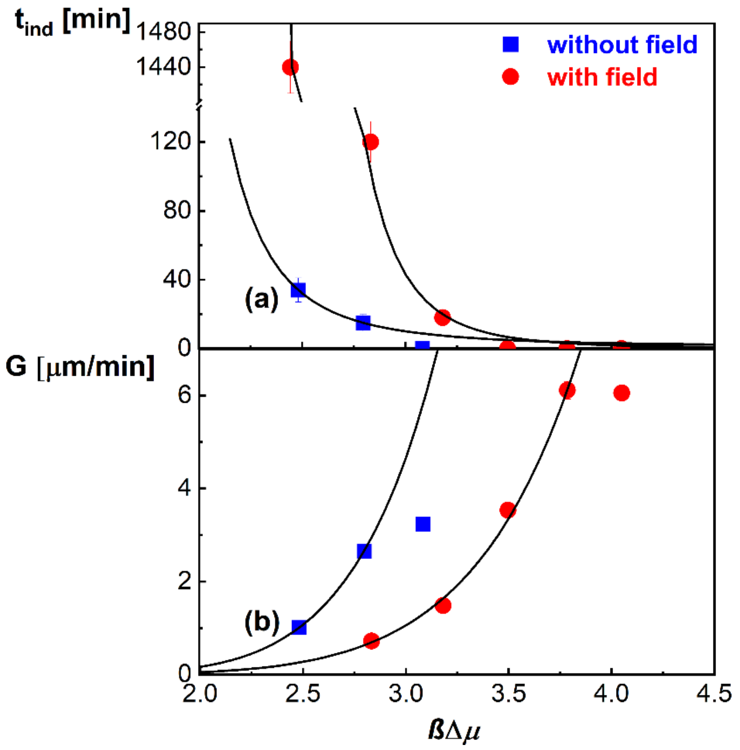

Fig S6. The dependence of (a) the crystallization induction time t_{ind} and (b) the initial crystal growth rate G on the chemical potential difference $\Delta\mu$. Data in the absence and presence of the electric field (symbols) and theoretical fits (lines) as discussed below.

Nucleation kinetics: Classical nucleation theory (CNT) provides a simple theoretical framework to describe nucleation as a thermally activated process. According to CNT, the nucleation rate J scales as

$$J \sim \exp(-\beta \Delta G^*),$$

where ΔG^* is the nucleation barrier which is given by

$$\Delta G^* = \frac{16}{3} \pi v_0^2 (\beta\gamma)^3 \frac{1}{(\beta\Delta\mu)^2}$$

with the volume of a protein in the crystal, assumed to have the literature value $v_0 = 30\ \mathrm{nm}^3$ [8] and the surface energy γ [7]. Thus, using $t_{ind} \sim J^{-1}$, the non-zero data in Fig. S6(a) could be described, yielding

$$\gamma = 0.34 \frac{kT}{R^2} \text{ ,}$$

$$\gamma = (0.47 \pm 0.02)\frac{kT}{R^2},$$

in the absence and presence of the electric field, respectively. Here, kT is the thermal energy and $R = 1.7$ nm is the typical radius of a lysozyme molecule. Similar values, 0.31 [7] and 0.36-0.46 [8] as expressed in the units used here, have been found in the literature.

Growth kinetics: The growth of lysozyme crystals can be described by a layer-by-layer model in which growth occurs as a 2D nucleation on the crystal surface [9,10]. The growth proceeds via the generation of steps followed by the incorporation of growth units (birth-and-spread model). Within this model, the growth rate normal to the crystal surface is given by

$$G = A\,(\beta\Delta\mu)^{1/6} \exp\left(\frac{2}{3}\beta\Delta\mu\right)\,[\exp(\beta\Delta\mu) - 1]^{2/3}\,\exp\left(-\frac{\pi}{3}\beta\tilde{\gamma}^2\,\frac{1}{\Delta\mu}\right).$$

The (effective) growth barrier is $\tilde{\gamma}$. The prefactor A is related to the attempt frequency at which the molecule tries to overcome the barrier. The effective barrier can be linked to the surface free energy of a step α via

$$\tilde{\gamma} = \alpha\sqrt{h\,v_0}$$

with step height h, set to 3 nm as in [8]. Excluding the data point with largest $\Delta\mu$, where G seems to reach a plateau and $t_{\mathrm{ind}} \sim 0$, we obtain:

$$\alpha = 1.0\,\frac{kT}{R^2},$$

$$\alpha = (0.91 \pm 0.06)\frac{kT}{R^2},$$

in the absence and presence of the electric field, respectively. These values are similar to those observed in [8].

3. References

[1] F. Platten, J. Hansen, D. Wagner, S.U. Egelhaaf, J. Phys. Chem. B 119, 14986 (2015).

[2] K. Kang, Rev. Sci. Instrum. 82, 053903 (2011).

[3] K. Kang, J.K.G. Dhont, Soft Matter 6, 273 (2010).

[4] J.-P. Guilloteau, M.M. Riès-Kautt, A.F. Ducruix, J. Cryst. Growth, 122, 223 (1992).

[5] M. Muschol, F. Rosenberger, J. Chem. Phys. 107, 1953 (1997).

[6] J. Hansen, S.U. Egelhaaf, F. Platten, Phys. Chem. Chem. Phys. 25, 3031 (2023).

[7] J. Drenth, K. Dijkstra, C. Haas, J. Leppert, O. Ohlenschläger, J. Phys. Chem. B 107, 4203 (2003).

[8] O. Galkin, P.G. Vekilov, J. Phys. Chem. B 103, 10965 (1999).

[9] H. Hondoh, T. Nakada, Cryst. Growth Design, 8, 4262 (2008).

[10] S. Gorti, E.L. Forsythe, M.L. Pusey, Cryst. Growth Design 4, 691 (2004).
