# Supplementary material for: The Effects of Electric Fields on Protein Phase Behavior and Protein Crystallization Kinetics
Source: J Phys Chem Lett. 2024 Aug 1;15(31):8108–13. doi: 10.1021/acs.jpclett.4c01744 (PMC11318033; doi:10.1021/acs.jpclett.4c01744)
Supplement: Supplementary file 2 — jz4c01744_si_002.pdf [file jz4c01744_si_002.pdf]

jz-2024-01744a.R1

Name: Peer Review Information for "The Effects of Electric Fields on Protein Phase Behavior and Protein Crystallization Kinetics"

First Round of Reviewer Comments

Reviewer: 1

Comments to the Author

In this manuscript the authors report about the impact of a (rather weak) AC field on the protein phase diagram (homogeneous phase vs. crystalline phase vs. liquid-liquid phase separation - LLPS), traced out in the protein vs. salt concentration plane. Throughout data are shown in the presence and the absence of the electric field. Further the crystallization dynamics is quantified by measuring the time-dependent length of the crystal and the transition into the LLPS regime is interpreted via a simple model. The present study represents a first, systematic study of these phenomena, using - in contrast to previous, related contributions - a rather weak field.

The manuscript is written in an extremely clear manner and it was a particular pleasure to read it. An extensive supplementary information is provided. The manuscript should by all means be published in the Journal of Physical Chemistry Letters; from my point of view only a few issues should be considered by the authors for the final version:

* The authors consider a particular value for the strength of the electric field; could the authors provide a qualitative outlook how their observations (shift in the transition lines, dependence of

\Gamma, dependence of \tau, etc.) might change as the field is modified moderately?

* the authors use the sticky hard sphere model for the interpretation of the transition into the LLPS regime; can the authors comment to what extent the assumption of spherical sphericity of the protein at hand is justified; again (and on a qualitative level): what can be expected when using a different (possibly strongly aspherical) protein?

* as for the sticky hard sphere model: (i) it might be helpful to provide a reference for the virial expansion of the osmotic pressure; (ii) the authors should be more specific about the impact of the stickiness parameter \tau: the current formulation ('... stickiness parameter \tau is a measure for the degree of short-ranged attractive interactions...') is too vague. This issue becomes crucial in view of the last phrase on page 4: 'The stickiness parameter is larger ...., indicating an apparent diminishing of the overall, ...attractive ... forces'. Does that mean: the larger \tau the weaker the potential?

* is the parameter t_ind a fitting parameter or is it taken from the experimental curves (e.g. from the inset in Fig. 2);

* Fig. 3: it might be helpful to indicate also the phase boundaries for the case that a field is applied;

* Fig. 4: the phrase 'The data points in Figure 4 correspond to ...' is somewhat vague; could the authors be more quantitative what protein concentration they consider; it might also be instructive to show the phase boundary (both with and without field) in this figure.

Reviewer: 2

Comments to the Author

This letter describes the effects of an alternating current (AC) electric field on the phase behavior of model protein lysozyme in the presence of sodium thiocyanate (NaSCN). The authors observed crystallization and liquid-liquid phase separation (LLPS) of lysozyme by optical microscopy with and without the AC electric field. Then, the authors found that the AC electric field moves the solution-crystal phase boundary in the direction of lower NaSCN concentration and moves the crystal-LLPS phase boundary in the direction of higher NaSCN concentration. The authors also found that the AC electric field accelerates the dynamics of the nucleation and growth of lysozyme crystals. The authors expect that the acceleration of the nucleation and growth dynamics is due to the increase in the anisotropic attractive interaction between lysozyme molecules. Then, the authors also speculate that the movement of the crystal-LLPS phase boundary is caused by the decrease in the overall orientationally averaged attractive force between lysozyme molecules.

The topics described in this paper are interesting from the viewpoints of physical chemistry and crystal growth. In addition, this letter is well written. Therefore, I recommend the publication of this letter in J. Phys. Chem. Lett. after the following minor revisions.

1) Abstract: The authors should explain that an AC electric field was used in this study.

2) Page 1, right column, lines 23-55: the authors should clearly explain what kind of things were new in this study from the experimental viewpoints because there are many previous studies on the effects of electric field. For example, the authors should explain how the conditions (V/mm and kHz) adopted in this study are mild compared to the previous studies (kV/mm and MHz).

3) Page 2, right column, lines 24-43: the authors should explain the crystal system of the authors' lysozyme crystals (orthorhombic?).

4) Page 4, left column, lines 6-8: The authors wrote that the nature of the driving force for the crystallization is not affected by the electric field. I could not understand why the authors could obtain such a conclusion from the contents shown in lines 1-5. The authors should add explanations.

Author's Response to Peer Review Comments:

Heinrich-Heine-Universität Düsseldorf 🖂 40204 Düsseldorf
WE Physik / Dr. F. Platten

Mathematisch-Naturwissenschaftliche Fakultät
WE Physik

Dr. Florian Platten

Telefon 02118112614
florian.platten@hhu.de

Düsseldorf, 25.07.2024

Heinrich-Heine-Universität Düsseldorf
Universitätsstraße 1
40225 Düsseldorf
Gebäude 25.31
Ebene U1 Raum 05

www.hhu.de

Dear Editor,

Thank you very much for sending the two referee reports on our manuscript jz-2024-01744a entitled “The Effects of Electric Fields on Protein Phase Behavior and Protein Crystallization Kinetics”.

Both referees were positive about the manuscript and recommend it for publication subject to minor revisions. We would like to thank the reviewers for their detailed comments which helped to improve the clarity of our manuscript. We have carefully considered all their suggestions and revised our manuscript accordingly. Please, find below our detailed response.

We also provide a detailed response to the manuscript formatting request.

We hope that our manuscript is now suitable for publication in *JPC Letters*.

With best regards,
Florian Platten and Kyongok Kang
(on behalf of all authors)

Response to Reviewer #1:

"In this manuscript the authors report about the impact of a (rather weak) AC field on the protein phase diagram (homogeneous phase vs. crystalline phase vs. liquid-liquid phase separation - LLPS), traced out in the protein vs. salt concentration plane. Throughout data are shown in the presence and the absence of the electric field. Further the crystallization dynamics is quantified by measuring the time-dependent length of the crystal and the transition into the LLPS regime is interpreted via a simple model. The present study represents a first, systematic study of these phenomena, using – in contrast to previous, related contributions - a rather weak field.

The manuscript is written in an extremely clear manner and it was a particular pleasure to read it. An extensive supplementary information is provided. The manuscript should by all means be published in the Journal of Physical Chemistry Letters; from my point of view only a few issues should be considered by the authors for the final version:"

> *Authors reply:*
> We thank the reviewer for the careful evaluation of our work and for the supportive feedback.

"* The authors consider a particular value for the strength of the electric field; could the authors provide a qualitative outlook how their observations (shift in the transition lines, dependence of \Gamma, dependence of \tau, etc.) might change as the field is modified moderately?"

> *Authors reply:*
> We agree with the referee and have added the following sentence to the end of the Conclusion:
> "In addition, future experiments will be carried out at various field conditions in order to examine whether the observed effects can be enhanced by the field amplitude and in order to decipher the role of different time scales by probing various field parameters."

"* the authors use the sticky hard sphere model for the interpretation of the transition into the LLPS regime; can the authors comment to what extent the assumption of spherical sphericity of the protein at hand is justified; again (and on a qualitative level): what can be expected when using a different (possibly strongly aspherical) protein?"

> *Authors reply:*
> Regarding its overall shape, lysozyme closely resembles a prolate ellipsoid with an axial ratio of about 1.5; i.e., it is roughly spherical (see, e.g., new Ref. [55]).
> With respect to the protein-protein interactions, the structure factor of lysozyme solutions under similar conditions also closely resemble those of the sticky sphere model.
> The extended law of corresponding states implies that, close to LLPS,

various models should be equivalent in terms of second virial coefficient. The sticky sphere model is likely the simplest model with short-ranged attractions, and has been previously applied to the LLPS of proteins. Therefore, the choice appears to be reasonable. These aspects are now briefly mentioned on p. 4:
"This is likely the simplest model for a system with short-range attractons and has been successfully applied to proteins [55]. Moreover, the corresponding-states law [56] implies that the model chosen should not matter on the second virial level."
It will be very interesting to see in future experiments whether this is also true for highly asymmetric proteins, such as antibodies. Comments in this regard would be speculative and are beyond the scope of the present work.

"* as for the sticky hard sphere model: (i) it might be helpful to provide a reference for the virial expansion of the osmotic pressure; (ii) the authors should be more specific about the impact of the stickiness parameter \tau: the current formulation ('... stickiness parameter \tau is a measure for the degree of short-ranged attractive interactions...') is too vague. This issue becomes crucial in view of the last phrase on page 4: 'The stickiness parameter is larger ...., indicating an apparent diminishing of the overall, ...attractive ... forces'. Does that mean: the larger \tau the weaker the potential?"

Authors reply:
(i) Details on the virial expansion can be found in standard textbooks on Statistical Thermodynamics; the reader is now referred to McQuarrie (new Ref. 57).
(ii) Indeed, this means that larger tau corresponds to weaker attractions. To further introduce the sticky sphere model, the following sentence is added on p. 4: "The sticky sphere model corresponds to a square well system whose well is infinitely narrow and deep in such a way that the second virial coefficient is finite [54]."

"* is the parameter t_ind a fitting parameter or is it taken from the experimental curves (e.g. from the inset in Fig. 2);"

Authors reply:
The respective sentence in the manuscript has been rephrased as follows: "the fitting parameters Γ and t_{ind} are the overall crystal growth rate and the induction time, respectively."

"* Fig. 3: it might be helpful to indicate also the phase boundaries for the case that a field is applied;"

Authors reply:
The boundaries in the presence of the field (at about 0.07 M and 0.15 M) are not shown for clarity.

“* Fig. 4: the phrase 'The data points in Figure 4 correspond to ...' is somewhat vague; could the authors be more quantitative what protein concentration they consider; it might also be instructive to show the phase boundary (both with and without field) in this figure.”

Authors reply:
The phase boundaries from Fig. 1 are now also indicated in Fig. 4, and the respective sentence now reads:
“The data points in **Figure 4** correspond to numerical values for the protein concentration at the LLPS phase boundary in **Figures 1b,c**.”

Response to Reviewer #2:

"This letter describes the effects of an alternating current (AC) electric field on the phase behavior of model protein lysozyme in the presence of sodium thiocyanate (NaSCN). The authors observed crystallization and liquid-liquid phase separation (LLPS) of lysozyme by optical microscopy with and without the AC electric field. Then, the authors found that the AC electric field moves the solution-crystal phase boundary in the direction of lower NaSCN concentration and moves the crystal-LLPS phase boundary in the direction of higher NaSCN concentration. The authors also found that the AC electric field accelerates the dynamics of the nucleation and growth of lysozyme crystals. The authors expect that the acceleration of the nucleation and growth dynamics is due to the increase in the anisotropic attractive interaction between lysozyme molecules. Then, the authors also speculate that the movement of the crystal-LLPS phase boundary is caused by the decrease in the overall orientationally averaged attractive force between lysozyme molecules.
The topics described in this paper are interesting from the viewpoints of physical chemistry and crystal growth. In addition, this letter is well written. Therefore, I recommend the publication of this letter in J. Phys. Chem. Lett. after the following minor revisions. "

> *Authors reply:*
> We thank the reviewer for the careful evaluation of our work and the positive feedback.

"1) Abstract: The authors should explain that an AC electric field was used in this study."

> *Authors reply:*
> This aspect is now mentioned in the Abstract.

"2) Page 1, right column, lines 23-55: the authors should clearly explain what kind of things were new in this study from the experimental viewpoints because there are many previous studies on the effects of electric field. For example, the authors should explain how the conditions (V/mm and kHz) adopted in this study are mild compared to the previous studies (kV/mm and MHz). "

> *Authors reply:*
> Indeed, this is an important point, and therefore the manuscript already contains many related explanations, as detailed below.
> In the second paragraph of the Introduction, we have already mentioned:
> "Electric fields have been employed to heuristically optimize the nucleation and growth processes to obtain diffraction-quality crystals, typically using high field amplitudes (kV/mm) or frequencies (MHz)."
> In this paragraph, we have now added:
> "However, in most of these studies, the electric field conditions are not very well defined."
> In the third paragraph, we have already mentioned:

"For the high electric field strengths used in many of the above-mentioned references, dielectric polarization plays an important role. (…) However, it is conceivable that for smaller field strengths, typically applied to colloids [35,36], other mechanisms might be dominant, which has not been systematically investigated for proteins."
In the last paragraph, we now explicitly mention:
"a relatively weak electric field (V/mm and kHz)"
In addition, this aspect has already been mentioned in the summary:
"The field conditions (6 V/mm and 1 kHz) are quite mild in comparison to earlier studies (typically kV/mm and MHz),"

"3) Page 2, right column, lines 24-43: the authors should explain the crystal system of the authors' lysozyme crystals (orthorhombic?). "

Authors reply:
As already mentioned in the Supporting Information, it is now stated on p. 2: "crystals in monoclinic form".

"4) Page 4, left column, lines 6-8: The authors wrote that the nature of the driving force for the crystallization is not affected by the electric field. I could not understand why the authors could obtain such a conclusion from the contents shown in lines 1-5. The authors should add explanations."

Authors reply:
To clarify, the respective sentence has been rewritten as follows:
"The same functional form of the growth curves indicates that the mechanism for crystallization and hence the driving force for crystallization is not affected by the electric field. That is, the effect of the electric field can therefore be understood entirely in terms of direct interactions between the proteins (…)"

Manuscript Formatting Request:

"1. An Abstract, which should summarize the reason for the work, the most significant results, and the conclusions, must be present and labeled."

Authors reply:
An abstract has already been provided and indicated in the Main Text: "We experimentally study the effect of an externally applied electric field on protein crystallization and liquid-liquid phase separation (LLPS) and the crystallization kinetics. For a surprisingly weak alternating current (AC) electric field, crystallization is found to occur in a wider region of the phase diagram, while nucleation induction times are reduced and crystal growth rates are enhanced. LLPS on the contrary, is suppressed, which diminishes the tendency for a two-step crystallization scenario. The effect of the electric field is ascribed to a change of the protein-protein interaction potential."

"2. The TOC graphic should fit in an area no larger than 3.25 in. × 1.75 in. (approx. 8.25 cm × 4.45 cm) and should have adequate resolution and clarity. Confirm that all text is legible at this size."

Authors reply:
A TOC graphic of proper size and with legible text has already been provided. The file is labelled as "toc.tif".

"3. Please provide a brief, nonsentence description of the actual contents of each Supporting Information file."

Authors reply:
A description of the content of the SI file has already been included in the Main Text:
"Supporting Information Available: Details on Materials & Methods and Supporting Data."
